# From Etiology to Aesthetic Surgery: Mapping Knowledge Domains and Emerging Trends in Gynecomastia Research: 35-Year Bibliometric Analysis

**DOI:** 10.1007/s00266-026-06027-3

**Published:** 2026-07-07

**Authors:** Kexin Deng, Yi Liu, Yu Tang, Ning Xie

**Affiliations:** 1 Department of Breast Cancer Medical Oncology, Hunan Cancer Hospital/The Affiliated Cancer Hospital of Xiangya School of Medicine, Central South University, No. 283, Tongzipo Road, Changsha, 410013 Hunan China; 2 Department of Plastic Surgery, The Third Xiangya Hospital, Central South University, Changsha, 410013 Hunan China; 3 Department of Oncology, Hunan Institute of Schistosomiasis Control (The Third People’s Hospital of Hunan Province), Yueyang, 414000 China

**Keywords:** Bibliometrics, CiteSpace, Plastic surgery, Gynecomastia, Reconstruction, Visualization

## Abstract

**Background:**

Gynecomastia is a benign proliferation of glandular tissue in the male breast, with incidence rate of 30–60% among adolescents. Its etiology involves physiological hormonal fluctuations, pathological conditions, and pharmacologic factors. It causes psychological burdens like body dysmorphic disorder and anxiety. Traditional reviews can’t synthesize numerous fragmented evidence, making objective and quantitative analysis needed.

**Method:**

The Web of Science Core Collection (SCIE) was data source to search literature about "gynecomastia" from 1990 to December 2024. After PRISMA process and triple-blind screening by three plastic surgery doctors, 863 English original articles were included. Software like CiteSpace, VOSviewer, and Excel were used for bibliometric and visual analysis.

**Result:**

Research showed three-stage stepwise growth trend. The United States was core of research (203 publications, betweenness centrality of 0.37). High-productivity institutions were mostly comprehensive universities and specialized hospitals. Author collaboration was decentralized. Core journals focused on plastic surgery and endocrinology fields. 12 keyword clusters were identified. Clusters with latest average years were #0 chest remodeling, #8 androgen receptor, and #9 dietary supplements. The ongoing hot issues include "risk factors,” "minimally invasive surgery,” "endoscopic treatment,” "hair removal,” "crystallized phenol,” "quality of life,” "explanation minimally invasive treatment,” and "efficacy.”

**Conclusion:**

This study visually presents research landscape and developmental trends in the gynecomastia field since 1990. Future research should focus on cases about elderly population, establish multidisciplinary teams (MDT), strengthen real-world studies, resolve conflicts between individual experiences, esthetics, and traditional social gender norms, and explore new technologies’ application like 3D printing and AI-assisted analysis.

**Level of Evidence III:**

This journal requires that authors assign a level of evidence to each article. For a full description of these Evidence-Based Medicine ratings, please refer to the Table of Contents or the online Instructions to Authors. www.springer.com/00266.

**Supplementary Information:**

The online version contains supplementary material available at https://doi.org/10.1007/s00266-026-06027-3 .

## Introduction

Gynecomastia refers to the benign proliferation of glandular tissue in the male breast, which is the most prevalent developmental anomaly of this organ [1]. From a pathological perspective, the condition is characterized by hyperplasia of both glandular epithelium and stromal components, which is distinct from adipose tissue accumulation alone [2, 3]. Although this condition can occur at any age, its peak incidence occurs during adolescence, affecting 30% to 60% of this population [4, 5]. The etiology is multifactorial, encompassing physiological hormonal fluctuations (e.g., estrogen–androgen imbalance in puberty), pathological states (hypogonadism, neoplasms, hepatic/renal dysfunction), and pharmacologic agents (antiandrogens, proton pump inhibitors) [6, 7].

The clinical manifestations typically present as unilateral or bilateral breast enlargement, frequently accompanied by a palpable discoid or nodular subareolar mass [2, 8]. Patients commonly report breast tenderness or pain upon palpation, and a few may experience nipple discharge. Although gynecomastia is a benign disorder, its detrimental impact on psychosocial functioning substantially exceeds that of physical discomfort [9]. There is evidence suggesting that the appearance of the breast can trigger clinically significant body dysmorphic disorder (BDD), manifested as anxiety, social avoidance, and depression [10]. Adolescent patients demonstrate diminished self-esteem and a tendency to be bullied [11], and their occupational impacts include military service restrictions [12]. This “psychological burden” represents the primary motivation for seeking intervention. Due to the increasing societal focus on masculine physique and the advancement of surgical techniques, contemporary management paradigms have gradually evolved from a purely medical approach to integrated functional–esthetic restoration. During this evolution, plastic surgery now occupies a key position [13].

Due to technological progress and heightened patient esthetic expectations, the treatment strategies for plastic surgery have significantly diversified [14]. The evolution has advanced from traditional open glandular resection to liposuction-assisted techniques [15], and ultimately to combined liposuction and minimally invasive gland excision [16]. Clinical studies in the included literature have reported that these minimally invasive technological advancements are associated with reduced scarring, accelerated postoperative recovery, and lower risks of complications such as hematoma and contour irregularity, which is consistent with the continuous research hotspot of 'minimally invasive surgery' identified in our bibliometric analysis [17]. Endoscopic surgery performed through the axilla and areola is consistently associated in the literature with the deepening of minimally invasive concepts and the pursuit of superior esthetic outcomes [18, 19]. Modern surgery research increasingly emphasizes functional–esthetic integration, encompassing comprehensive body contouring, structural restoration, and artistic design, reflecting a shift toward multidisciplinary perspectives.

However, significant gaps still exist: there is no standardized assessment tool for evaluating the severity of gynecomastia and the effectiveness of treatment, and there is a lack of unified clinical standards for various treatment options. The traditional assessment methods clearly cannot effectively integrate those scattered and numerous pieces of evidence, thereby impeding clinical optimization and academic progress. Bibliometrics compensates for these deficiencies through its objective, quantitative, and visual methodology [20, 21]. By mapping knowledge architectures, research fronts, and developmental trends, this approach provides a comprehensive perspective to reveal disciplinary patterns and guide future research trajectories [22, 23].

## Method

### Research Methods

In this study, CiteSpace 6.4.R1(64-bit) Advanced, VOSviewer 1.6.18, and Microsoft Excel 2021 were used for bibliometric analysis and visualization [24–30]. The specific analysis procedures and parameter settings are as follows:

1. Descriptive statistical analysis: Microsoft Excel 2021 was used to conduct descriptive statistics on the annual publication volume, country/institution/author publication output, and journal distribution, to describe the overall development trend of gynecomastia research.
2. Collaboration network analysis: CiteSpace 6.4.R1 was used to construct the collaboration network of countries, institutions, and authors. The key parameter settings were: time slicing from 1990 to 2024 with 1 year per slice; term source including title, abstract, author keywords, and keywords plus; selection criteria set as g-index (k=25); the pathfinder pruning algorithm was used to optimize the network structure. Betweenness centrality was calculated to measure the bridging role of each node in the network, with a value >0.1 indicating an important pivotal node.
3. Keyword co-occurrence and cluster analysis: CiteSpace 6.4.R1 was used for keyword co-occurrence analysis, and the log-likelihood ratio (LLR) algorithm was used for cluster labeling to identify the core thematic structure of the research field. The parameter settings were consistent with the collaboration network analysis, and the minimum cluster size was set to 10.
4. Keyword burst detection: The Kleinberg algorithm built into CiteSpace 6.4.R1 was used to detect keywords with a sharp increase in citation frequency, with the minimum burst duration set to 2 years, to identify emerging research hotspots and frontier trends.
5. Journal co-citation analysis: VOSviewer 1.6.18 was used for journal co-citation analysis and visualization, with the counting method set to full counting and the clustering resolution set to 1.0**.**

Then we investigated the key research outcomes and critical insights of this topic through critical reading. Detailed descriptions of the software and comprehensive information regarding the software are available in the supplementary files.

### Literature Screening and Quality Control

This study used the Web of Science Core Collection (WoSCC)—the Science Citation Index Expanded (SCIE)—as its sole data source to ensure the authority and quality of the included literature. The search query used in this paper is as follows: TS=(“gynecomastia*” OR “gyneacomastia*”), where TS represents the topic field, covering the title, abstract, author keywords, and keywords plus of the literature. The time span of the literature was strictly limited from January 1, 1990 to December 31, 2024, and the final electronic retrieval operation was completed on July 1, 2025.The complete retrieval record and original data have been archived. A total of 3,452 records were obtained in the initial search. The screening process was conducted in strict accordance with the PRISMA 2020 guidelines, with specific steps as follows:

Document type screening: A total of 1371 records were excluded, including non-English publications, reviews, letters, case reports, conference abstracts, editorial materials, retracted articles, and studies absolutely irrelevant to gynecomastia based on title and abstract.

Full-text screening: The remaining 2,081 articles were independently reviewed by three PhD-level researchers specializing in plastic surgery from different institutions independently in a blinded fashion A total of 1,218 articles that did not meet the inclusion criteria were excluded, including studies focusing on pseudogynecomastia, studies that only mentioned gynecomastia as a differential diagnosis or incidental finding, and studies with incomplete data; the specific screening criteria are detailed below.

Deduplication: Four duplicate records were removed using the built-in deduplication function of WoSCC and EndNote X9 software.

**Fig. 1:**
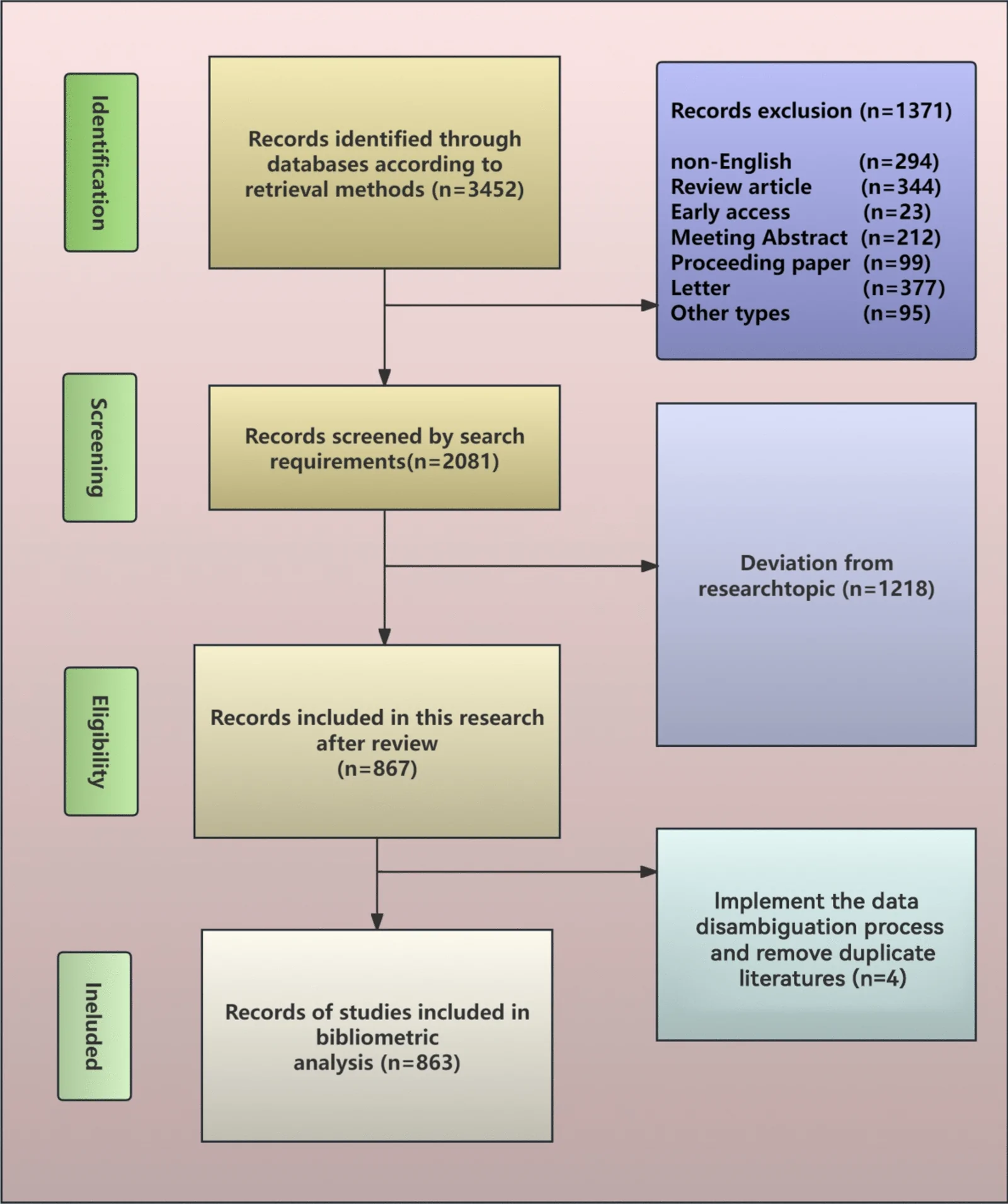
PRISMA flow diagram illustrating the search strategy employed in the current bibliometric study

Finally, 863 English original articles were included in the final bibliometric analysis. The inter-rater reliability (Cohen’s kappa coefficient) of the three independent reviewers was 0.92, indicating excellent consistency. Any discrepancies during the screening process were resolved through group discussion with a senior plastic surgery expert to ensure the accuracy and relevance of the included literature. The updated PRISMA flow diagram (Fig. 1) is fully consistent with the data described in the text.

#### Inclusion Criteria

1. The search was restricted to English-language publications with complete references and citation records.
2. The core content and research objective of the studies focused on Gynecomastia, which refers to the abnormal hyperplasia and development of male breast glandular tissue (rather than simple adipose tissue).
3. The main content of the studies covered various aspects, including the epidemiology of the disease, disease risk factors, pathology, diagnosis, differential diagnosis, management approaches, complications, genetic studies, comorbid diseases, and prognosis.
4. The research content was confined to human subjects.

#### Exclusion Criteria

1. Non-English literature and articles with incomplete references or citations.
2. Studies that primarily focus on Pseudogynecomastia, which is merely adipose tissue accumulation without breast glandular hyperplasia and commonly seen in obese males.
3. Studies where gynecomastia is merely mentioned as a differential diagnosis or an incidentally associated disease.
4. Studies on gynecomastia in other mammals (e.g., dogs, horses).
5. Articles that incidentally mention keywords related to gynecomastia but have research objectives or materials unrelated to these keywords.

## Results and Discussion

### Analysis of Publishing Trend

**Fig. 2:**
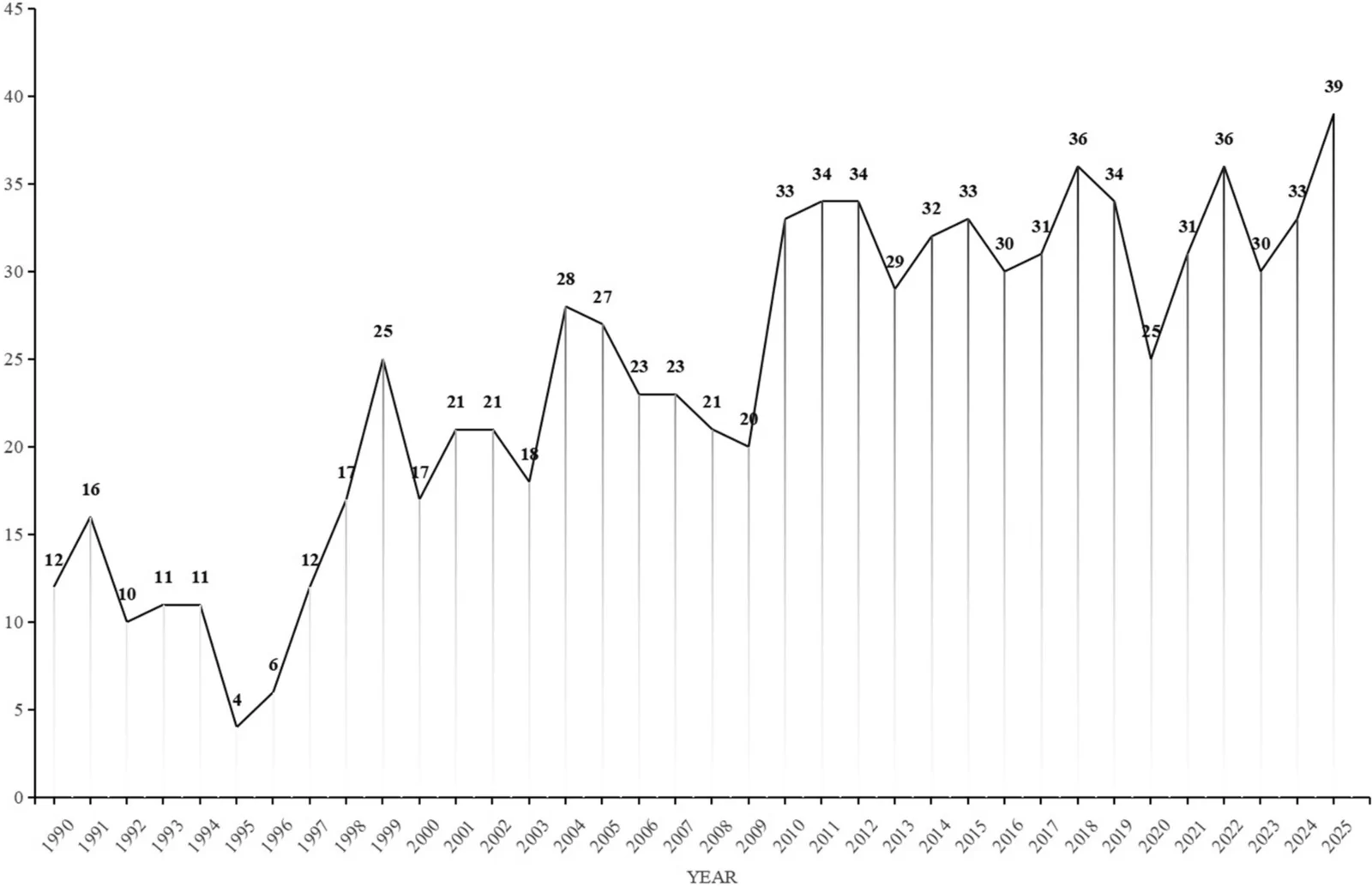
Curve of publication frequency changes over time in the field of gynecomastia

As shown in Fig. 2, the research is divided into three phases, showing a stepwise upward trend (Fig. 2). From 1990 to 1998, the number of studies was relatively small, approximately 11 each year. From 1999 to 2009, it was the second plateau period, with an average of 22 studies per year; this stepwise growth trend is temporally consistent with the development and clinical popularization of minimally invasive technology in plastic surgery, which may be one of the important driving factors for the continuous increase in research output in this field, as reflected in the subsequent keyword hotspot analysis. From 2010 to the present, this is the highest plateau period, with the number of studies stabilizing at 52 each year. Despite periodic fluctuations, the relevant literature still maintains stable growth, marking that this field has moved from exploratory research to the mature stage. With the improvement of socioeconomic conditions, the popularization of social media, and the increased attention to quality of life and mental health [31, 32], it is expected that the research literature related to gynecomastia will continue to maintain a strong growth momentum.

### National, Institutional, Author, and Journal Analysis

#### National Analysis

**Fig. 3:**
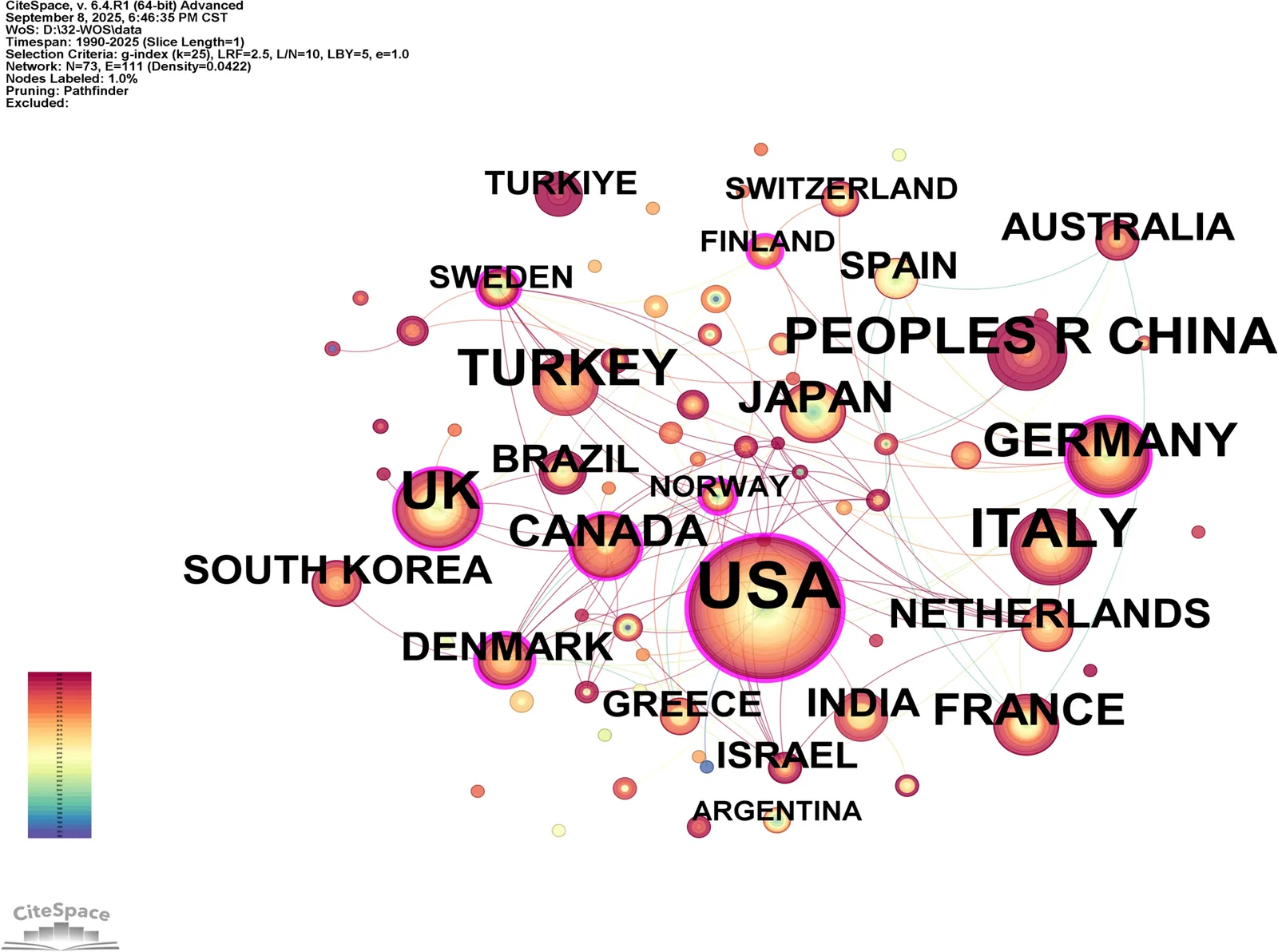
National collaboration network in the field of gynecomastia. Node size represents publication output volume. Chromatic coding of nodes and lines indicates publication years and collaborative timelines respectively. Nodes featuring purple peripheries denote intermediary centrality >0.1, identifying pivotal bridges within the collaborative framework

Analysis of the top 20 most contributing countries, as shown in Fig. 3 and Table 1, revealed different research patterns across regions. The United States, with robust international collaboration (centrality 0.37) and the highest publication output, has become the core hub in this field. Apart from the United States, the top 20 countries are mostly economically developed ones, such as Italy, Germany, France, Canada, the Netherlands, and the UK. This high-output pattern in developed countries is associated with their higher socioeconomic level, sufficient research investment, and higher attention to male esthetic medicine and mental health-related fields [33], which is consistent with the keyword cluster results of “appearance related burdens” and “quality of life”. Emerging economies like China, Turkey, India, and Brazil have effectively obtained large-sample clinical data through demographic advantages, driving clinical needs-focused innovation. It is notable that only the United States, the United Kingdom, and Denmark have centrality values higher than 0.1. The centrality values of other countries are lower than 0.1, which reflects the insufficient international cooperation and, to a certain extent, limits the level of research achievements and development.

**Table 1:** Top 20 countries with the highest publication counts

Country	Frequency	Intermediary Centrality	Earliest Publication Year
USA	203	0.37	1990
Italy	77	0.08	1991
UK	70	0.18	1993
People’s Republic of China	58	0	1999
Turkey	57	0	1992
Germany	41	0.06	1997
France	33	0.02	1998
Canada	33	0.05	2002
Japan	33	0	1990
The Netherlands	23	0.04	2002
India	21	0.04	2001
South Korea	21	0	2005
Denmark	21	0.1	1998
Brazil	20	0.05	1999
Australia	19	0.02	1998
Spain	17	0	1991
Israel	16	0.01	1991
Greece	15	0.04	1994
Egypt	13	0.08	2003
Poland	13	0	2007

#### Institutional Analysis

**Fig. 4:**
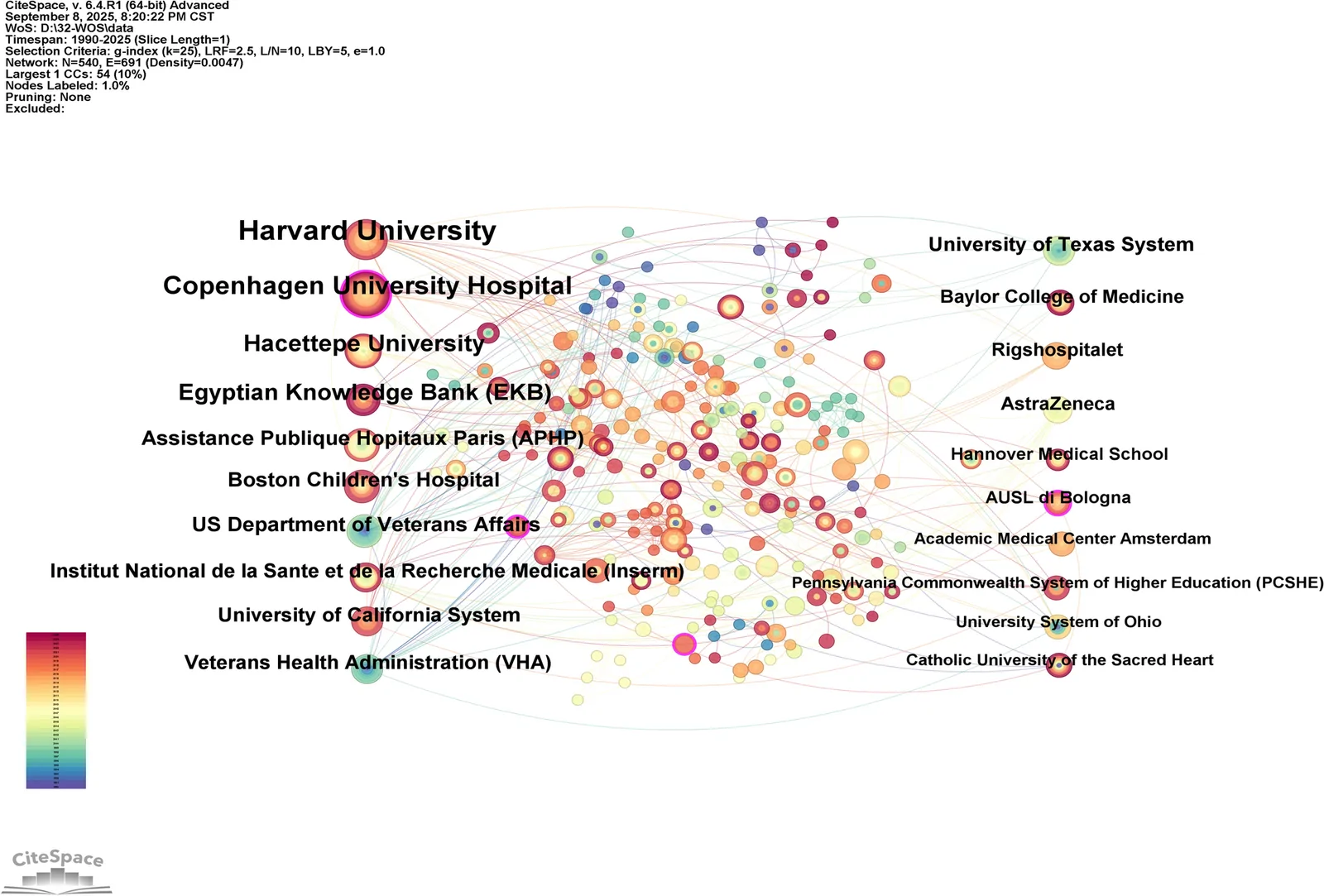
Institutional co-occurrence map. Using “Institution” as the node type in CiteSpace, we generated a co-occurrence map with 540 nodes, 691 links and a network density of 0.0047. Nodes represent academic institutions, with size proportional to publication output. Purple-ringed nodes denote entities exhibiting high brokerage potential (betweenness centrality >0.1) within the cooperative landscape

Institutions are taken as the analysis unit, and the top 20 contributing institutions are presented in Fig. 4 and Table 2**.** This group mainly consists of elite comprehensive universities and leading specialized hospitals. Exemplified by Harvard University and Hacettepe University—which integrate medical schools, affiliated hospitals, and comprehensive research systems—these institutions efficiently consolidate multidisciplinary resources and pathological materials. This integration enables multidimensional investigation from basic mechanisms to clinical therapies, generating high-output research outcomes. Furthermore, medical institutions like Copenhagen University Hospital, Assistance Publique-Hôpitaux de Paris (APHP), and Boston Children’s Hospital leverage extensive clinical case repositories and specialized teams to conduct research that informs clinical practice.

**Table 2:** Top 20 institutions with the highest publication counts

Institution	Frequency	Intermediary Centrality	Earliest Publication Year
Harvard University	24	0.09	1993
Copenhagen University Hospital	18	0.16	2003
Hacettepe University	14	0	1992
Egyptian Knowledge Bank (EKB)	13	0	2003
Assistance Publique Hopitaux Paris (APHP)	9	0.01	2001
Boston Children's Hospital	9	0	2006
US Department of Veterans Affairs	9	0.03	1990
University of California System	8	0.02	1993
Veterans Health Administration (VHA)	8	0.02	1990
Institut National de la Sante et de la Recherche Medicale (Inserm)	8	0.07	2002
University of Texas System	8	0.01	1995
Baylor College of Medicine	7	0	1998
Rigshospitalet	7	0	2012
AstraZeneca	7	0.1	2004
Hannover Medical School	6	0.03	1998
AUSL di Bologna	6	0.12	2007
Academic Medical Center Amsterdam	5	0	2012
Pennsylvania Commonwealth System of Higher Education (PCSHE)	5	0.02	1998
University System of Ohio	5	0.06	1990

It is noteworthy that the collaboration degree of most institutions is severely insufficient (centrality less than 0.01), although the connections between institutions are still crucial for maintaining academic progress. Research institutions should actively pursue resource sharing and cooperative synergy. The analysis of high-output institutions reveals the common advantages of different organizational types: integrated education-research models, multidisciplinary frameworks, robust data resources, and strong integration capacity.

#### Author Analysis

**Fig. 5:**
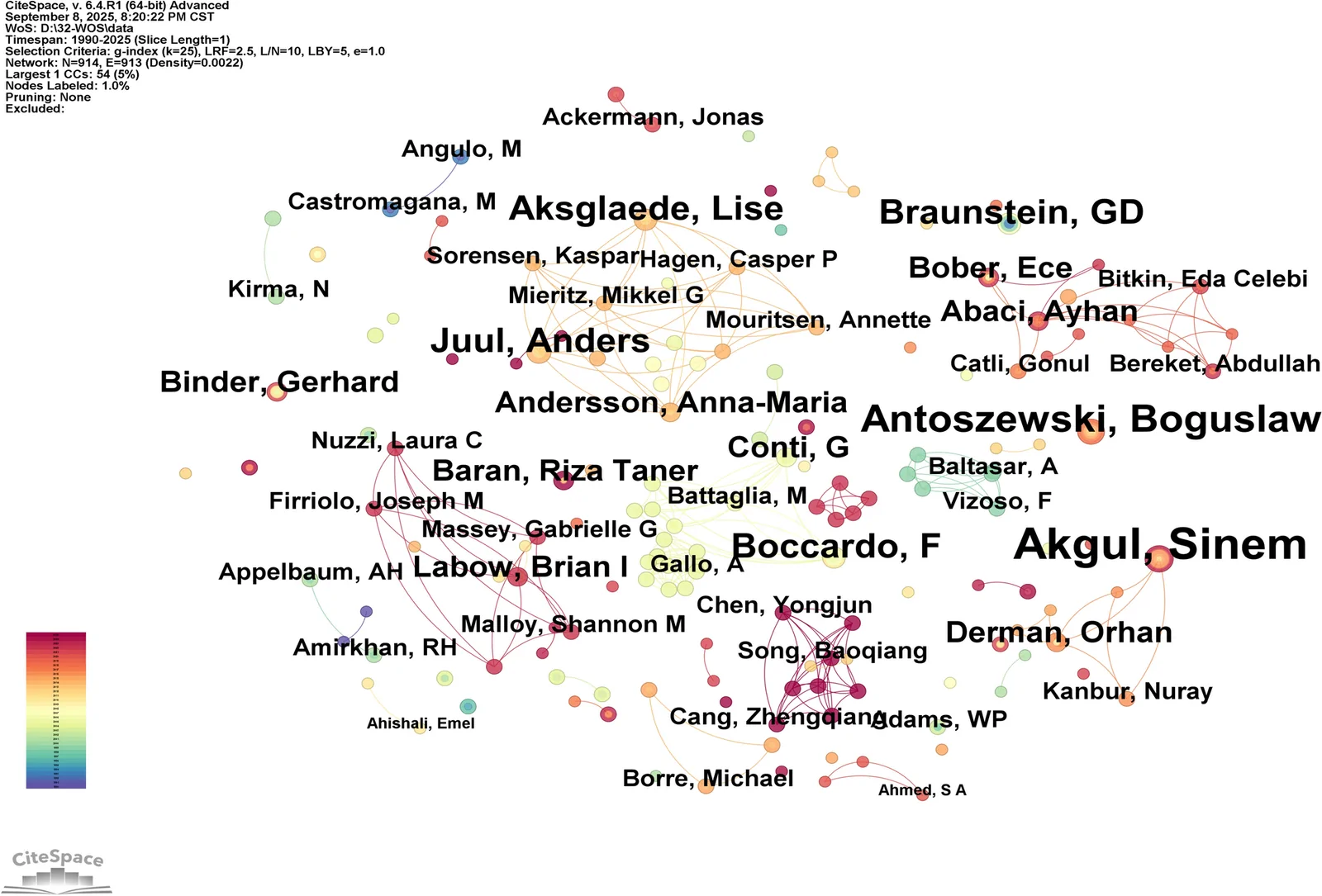
Analysis of authors' publication output and collaboration networks. Using “Author” as the node type in CiteSpace, we generated an author collaboration and publication network map. This graph includes 914 network nodes (with a node threshold set at over 4 publications), 913 connections, and a density of 0.0022. Node size corresponds to the number of articles each author has published, and connections between authors with limited collaborative engagement have been excluded

In Fig. 5 and Table 3, no individual author published more than 8 articles, and the most prolific contributor was Sinem Akgul, with 7 publications. All included authors exhibited a betweenness centrality of zero, reflecting a decentralized authorship pattern in this field, indicating substantial research independence. Regarding the comparatively low per capita publication output, we speculate that this may reflect the current research landscape of gynecomastia—characterized by a greater emphasis on clinical aspects than on multidisciplinary collaborative basic science, which is consistent with the low centrality results of most countries and institutions. Simultaneously, limited collaboration among authors likely constrains the pace of research output. Furthermore, we believe that gynecomastia-related privacy concerns could also be one of the key barriers to multicenter clinical collaborations.

**Table 3:** Authors with the highest publication counts

Author	Frequency	Centrality	Earliest Publication Year
Akgul, Sinem	7	0	2011
Antoszewski, Boguslaw	5	0	2012
Aksglaede, Lise	4	0	2011
Boccardo, F	4	0	2005
Braunstein, GD	4	0	1993
Juul, Anders	4	0	2012
Abaci, Ayhan	3	0	2017
Andersson, Anna-Maria	3	0	2012
Baran, Riza Taner	3	0	2014
Binder, Gerhard	3	0	2005
Bober, Ece	3	0	2005
Conti, G	3	0	2005
Derman, Orhan	3	0	2008
Labow, Brian I	3	0	2022
Ackermann, Jonas	2	0	2020
Adams, WP	2	0	1998
Al mufti, Ragheed	2	0	2008
Amirkhan, RH	2	0	1999
Angulo, M	2	0	1991
Appelbaum, AH	2	0	1999

#### Journal Analysis

**Fig. 6:**
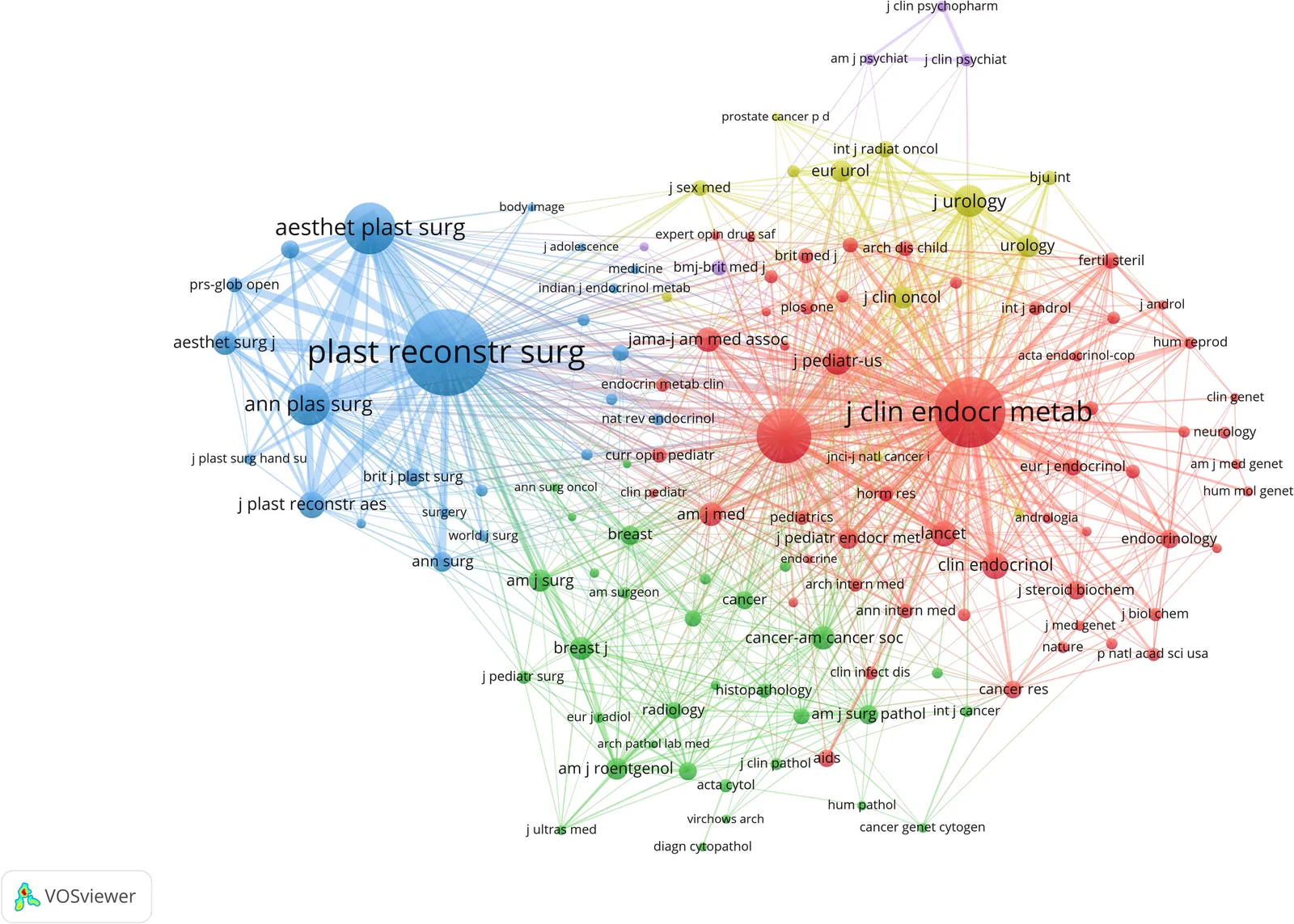
Analysis map of journal co-citation network. Node size corresponds to journal output (total number of publications), while color-based clustering reflects thematic resemblance of cited literature. Connections between nodes indicate co-occurrence in the reference lists of citing papers

As shown in Fig. 6 and Table 4, *Plastic and Reconstructive Surgery* ranks first with 1,312 citations, establishing its position as the core knowledge source in this field. This trend is also reflected in plastic surgery journals such as *Aesthetic Plastic Surgery* and *Annals of Plastic Surgery*, with their publication volume accounting for one-third of total output. This reflects the field’s primary focus on clinical modern gynecomastia treatments and technological innovation.

**Table 4:** Top 15 journals ranked by number of documents

Journal	Documents	Citations
Aesthetic Plastic Surgery	67	784
Annals of Plastic Surgery	39	738
Plastic and Reconstructive Surgery	34	1312
Journal of Clinical Endocrinology & Metabolism	21	1047
Journal of Pediatric Endocrinology & Metabolism	21	169
Aesthetic Surgery Journal	14	191
Andrologia	12	75
Journal of Plastic Reconstructive and Aesthetic Surgery	11	165
Breast Journal	11	125
Journal of Urology	10	502
Breast	10	175
Urology	8	269
European Journal of Endocrinology	7	118
New England Journal of Medicine	6	1318
Journal of Pediatric Surgery	6	166

By contrast, journals like the *New England Journal of Medicine* and *Journal of Clinical Endocrinology & Metabolism,* although not leading in terms of publication volume, achieve exceptional citation impact [34, 35]. This indicates that in the basic fields, such as the etiology of gynecomastia, pathophysiological mechanisms, and pharmacologic/non-surgical interventions, there exists high-quality and influential research.

Collectively, all the listed journals represent authoritative high-impact publications in their respective disciplines. They portray a research landscape where plastic surgery is at the forefront of clinical and technical advancements in pathophysiological mechanisms—a field characterized by distinct specialization yet profound multidisciplinary integration.

### Keywords Clusters

**Fig. 7:**
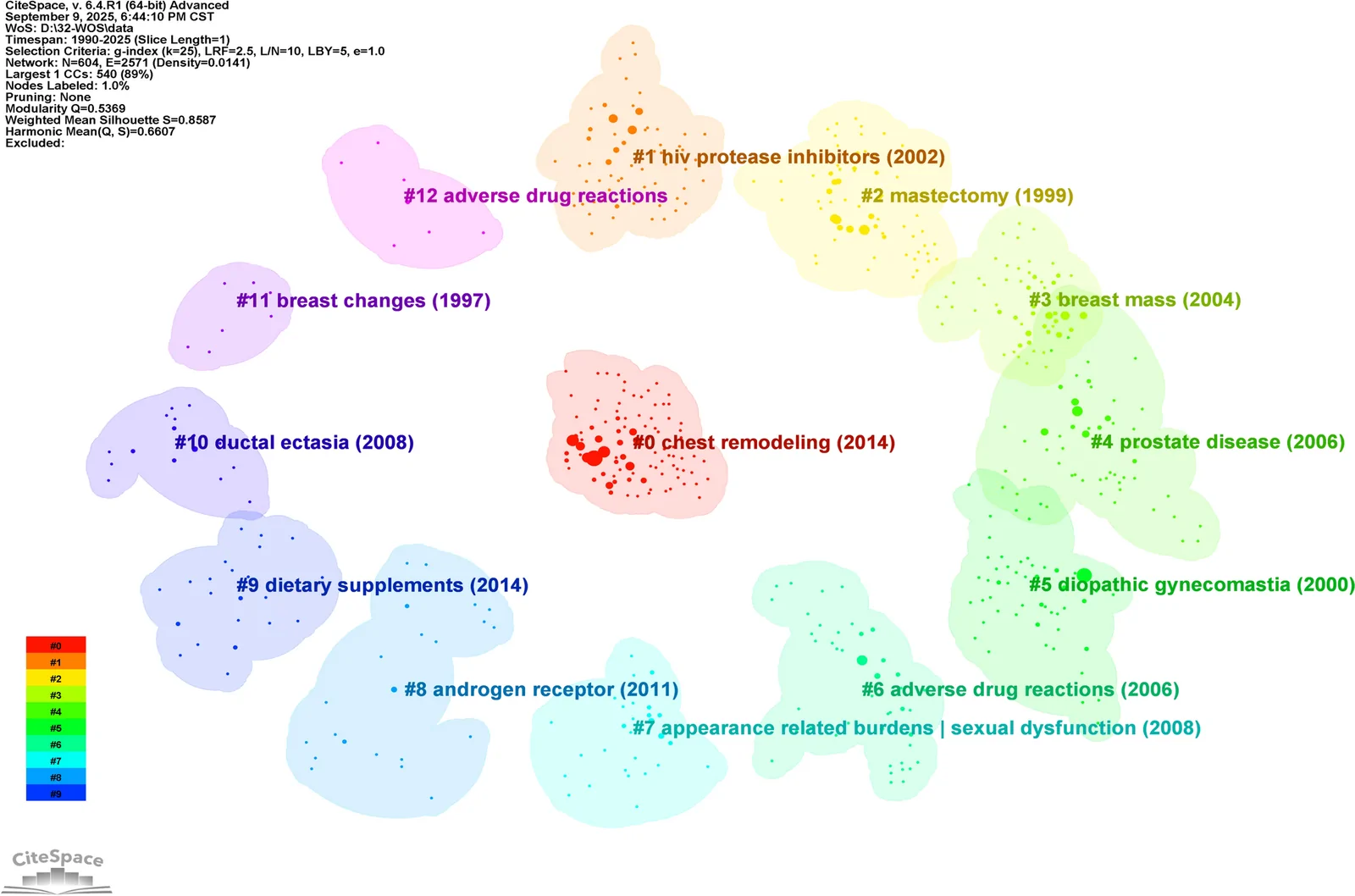
Cluster analysis. Cluster analysis conducted via CiteSpace's Pathfinder pruning algorithm identified 13 thematic groups. Cluster labels were extracted through LLR-based keyword saliency ranking, with internodes linkages optimized for network connectivity

These 12 clusters are shown in Fig. 7 as follows**:** #0 chest remodeling (2014), #1 hiv protease inhibitors (2002), #2 mastectomy (1999), #3 breast mass (2004), #4 prostate disease (2006), #5 idiopathic gynecomastia (2000), #6 adverse drug reactions (2006), #7 appearance related burdens | sexual dysfunction (2008), #8 androgen receptor (2011), #9 dietary supplements (2014), #10 ductal ectasia (2008), #11 breast changes (1997)

For the earlier keywords clusters #11, #4, and #6, their consistent research theme is iatrogenic or secondary gynecomastia. The specific manifestations are as follows:

The earliest cluster #11 focuses on drug-induced gynecomastia (e.g., keywords in the cluster such as “omeprazole therapy,” “potassium canrenoate,” “spironolactone,” “ketoconazole,” “cimetidine” that block testosterone synthesis) and cancer treatment-induced gynecomastia (e.g., keywords “blocks testosterone synthesis,” “cytotoxic therapy,” “testicular cancer”) [36]; Clusters #4 and #6 further expanded the research theme on cancer treatment-induced gynecomastia (e.g., keywords “prostate disease,” “androgen deprivation therapy,” “bicalutamide,” “tamoxifen,” etc.). In addition, concepts for preventing cancer treatment-induced gynecomastia such as “breast irradiation” and “prophylactic radiotherapy” have been proposed. Other drugs that can induce gynecomastia, such as “atorvastatin,” “doping,” “oestradiol,” and “congenital hypothyroidism,” have also received further attention [37].

It can be seen that secondary gynecomastia was the focus of early research. This type of gynecomastia has a high incidence in clinical practice.

The focus of Cluster #5 is “idiopathic gynecomastia,” which refers to gynecomastia with unknown causes after excluding clear inducing factors such as drugs, diseases, and hormonal abnormalities [38]. It accounts for approximately 25%−50% of clinical cases and is more common in post-adolescent or adult males [39, 40]. Research was conducted around core basic mechanisms such as “estrogen”–“androgens” imbalance, “pituitary testicular axis,” and “pituitary hormones,” making it the most important basic research cluster in the early stage.

The research focuses of Clusters #1, #3, #8, and #10 are the differential diagnosis, comorbid diseases, and associated diseases related to gynecomastia. Clusters #1 and #3 have always been dedicated to establishing methods to distinguish gynecomastia (benign) from male breast cancer (malignant) and other breast lesions, covering differentiation criteria at the levels of clinical signs, imaging examinations, pathophysiology, and genetic laboratories. This cluster has also been dedicated to revealing differences at the pathological morphology or molecular level. Relevant keywords include “heterogeneity” (diversity of pathological/molecular characteristics among lesions), “amplifications,” “amyloidosis,” “secreting macroadenomas,” and “sertoli cell tumors.”

The appearance of keywords such as “androgen receptor,” “androgen insensitivity syndrome,” “central precocious puberty,” “klinefelter syndrome,” and “endocrine adverse reaction” in Clusters #8 and #10 explains pathological gynecomastia from four dimensions: “gene-chromosome–hormone–drug,” covering the main etiological types of gynecomastia. Besides distinguishing gynecomastia from the most common breast cancer, differential diagnosis-related terms such as “ductal ectasia,” “neonatal mastitis,” “juvenile papillomatosis,” and “granular cell (lesions)” have also become the focus of further research in these clusters; “US findings,” “whole exome sequencing (WES),” and “46XY karyotype” are representative core tools in the clusters, which are used to achieve the above differentiations and ensure diagnostic accuracy [41, 42].

Around 2008, Cluster #7 analyzed the correlations between “appearance related burdens,” “sexual dysfunction,” “attitudes,” “ratings,” “ethics” and gynecomastia from a sociological perspective, focusing on the conflict between traditional gender norms and individual experiences. The core contradiction, “appearance related burdens,” was specifically manifested as self-stigmatization and social withdrawal [43]; “sexual dysfunction” was a common comorbidity in patients with gynecomastia [44]. For example, it was caused by keywords such as “estrogen,” “testicular steroid biosynthesis,” and “galactorrhea,” forming a physiological–social vicious cycle. “Attitudes” and “ratings” reflected the quantification of social acceptance and interpersonal interaction dilemmas faced by patients with gynecomastia. Combined with clinical trials, “ethics” needed to focus on sociological ethical issues in gynecomastia medical intervention, with the core lying in preventing medical practice from succumbing to social esthetic norms and safeguarding patients’ health rights and social dignity [45].

With average years ranging from the early 1990s to the recent 2014, Clusters #0, #2, and #9 have been focusing on the content related to gynecomastia surgical operations, mainly expanding around four core dimensions: surgical method classification, minimally invasive technology application, postoperative appearance optimization, and patient group adaptation. These are the eternal focuses in the entire field. Based on the basic surgical methods of “liposuction” + “subcutaneous mastectomy,” combined with “endosurgery” to reduce trauma; skin folding and chest reshaping were used to optimize postoperative appearance; simultaneously, individualized plans were formulated for different patient groups such as “adolescent” and “massive weight loss.”

### Keywords Outbreak Analysis

**Fig. 8:**
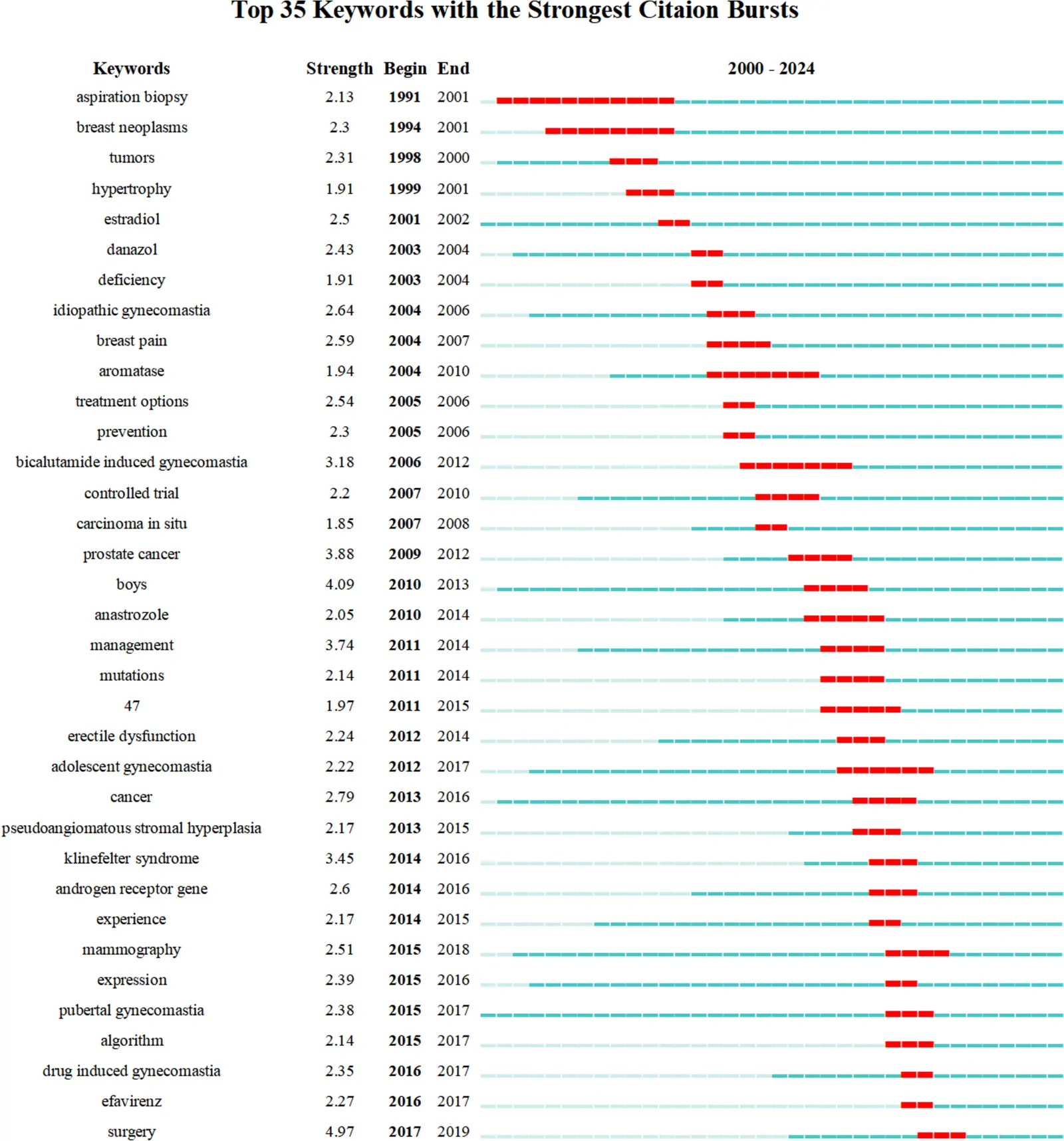
Top 35 keywords with the strongest citation bursts. The timeline is represented by a blue line, while the embedded red segments indicate the periods of heightened research activity for each keyword

In this study, the Bursts detection algorithm was applied in CiteSpace to visualize the evolution of research hot spots related to gynecomastia within the Web of Science database, as shown in Fig. 8. The generated map presents the top 35 keywords, illustrating their burst intensity and duration. Currently, the most significant keywords include “risk factors,” “minimally invasive surgery,” “endoscopic treatment,” “hair removal,” “crystallized phenol,” “quality of life,” “explanation minimally invasive treatment,” “efficacy.”

By sorting out the timeline of keyword bursts (1990–2025), we can clearly observe that the research field of gynecomastia has evolved through stages from basic diagnosis and treatment, to precise individualized diagnosis and treatment, and then to patient-centered treatment outcomes.

Phase 1 (1990–2003): Focus on malignant differentiation and basic diagnosis—addressing the top clinical priority of determining danger. Core burst keywords included “aspiration biopsy”, “breast neoplasm,” and “hypertrophy.”

Phase 2 (2001–2010): In-depth research on etiological mechanisms and drug treatments—meeting the core needs of understanding pathogenesis and medication strategies. Core burst keywords included “estradiol,” “aromatase,” “danazol,” “treatment options,” and “controlled trial.” This proves that in terms of treatment, the field has shifted from unregulated medication to evidence-based optimization.

Phase 3 (2010–2017): Research focusing on subpopulations and etiological subtypes and moving toward individualized diagnosis and treatment. Core burst keywords included “adolescent gynecomastia,” “klinefelter syndrome,” “androgen receptor gene,” “bicalutamide induced gynecomastia,” and “prostate cancer.”

These trends reflect the shift from generalized gynecomastia to subpopulations and etiological subtypes, indicating the clinical trend of individualized diagnosis and treatment.

Phase 4 (2015–2025): Focus on standardized diagnosis and treatment as well as patient outcomes—from treating diseases to treating the whole person.

The continuous bursts of “classification” (e.g., adipose, glandular, mixed types) marks the transition from “individualization” to “standardization.” Accurate classification reduces the differences in diagnosis and treatment among different medical institutions. In recent years, the frequent appearance of terms such as “plastic surgery,” “surgical treatment,” and “liposuction” is due to the maturity of minimally invasive surgical techniques (e.g., “endoscopic liposuction,” “subcutaneous mastectomy”). Surgery has become the main choice for patients, and the research focus has shifted to optimizing surgical methods. Meanwhile, the bursts of “patient satisfaction,” “complications,” and “outcom” marks the shift of the medical pattern from the biomedical pattern to the bio-psycho-social pattern. Attention is no longer limited to whether the glands have been removed, but also includes whether the postoperative appearance conforms to male esthetics, whether patients are satisfied, and whether there are complications such as scars/nipple necrosis, which reflects the emphasis on the quality of patients’ lives.

This study has certain limitations: first, we only included English original articles from the WoSCC database, which may have a certain degree of publication bias and language bias; second, bibliometric analysis can only objectively map the research landscape based on published literature, and cannot conduct in-depth evaluation of the methodological quality and evidence level of individual clinical studies.

## Conclusion

This article provides a visualized summary of the research on gynecomastia since 1990, describing the research landscape and framework in this field at the national, institutional, author, and journal levels, and mapping out the development trajectory of the research as well as the current hotspots and research trends.

Building on our findings, we put forward the following suggestions for future gynecomastia research and clinical translation:

1. Research should continuously focus on new clinical challenges, such as geriatric gynecomastia, obesity-related gynecomastia, and psychological intervention for adolescent gynecomastia.
2. The treatment of gynecomastia involves multiple departments, including endocrinology, surgery, radiology, pathology, genetics, and psychology. In the future, a multidisciplinary team (MDT) for gynecomastia should be established, especially for complex subtypes, to avoid the limitations of single-discipline diagnosis and treatment.
3. Field research on patients’ actual experiences should be strengthened, such as conducting long-term follow-up surveys on patients who have undergone surgery to evaluate their appearance satisfaction, psychological state improvement, and sexual life status, rather than merely conducting short-term evaluations of the rate of gland removal. At the same time, cost–benefit analysis should be conducted to provide a basis for medical insurance policies and marketization.
4. The applications of new technologies should be explored, such as 3D printing-assisted surgical planning to optimize liposuction scope, AI-assisted differential diagnosis, and laser-assisted skin tightening to improve postoperative skin laxity, thereby further improving the efficiency and quality of diagnosis and treatment.

These proposals are based on the research gaps identified by bibliometric analysis, rather than confirmed clinical conclusions

## Supplementary Information

Below is the link to the electronic supplementary material.

Supplementary material 1 (DOCX 20.2 kb)

## Data Availability

Not available.
