# Supplementary material for: From Etiology to Aesthetic Surgery: Mapping Knowledge Domains and Emerging Trends in Gynecomastia Research: 35-Year Bibliometric Analysis
Source: Aesthetic Plast Surg. 2026 Jul 7;50(17):7109–23. doi: 10.1007/s00266-026-06027-3 (PMC13631031; doi:10.1007/s00266-026-06027-3)
Supplement: Supplementary file 1 [file 266_2026_6027_MOESM1_ESM.docx]

**Supplementary material**

CiteSpace is an information visualization tool based on citation analysis theory developed by Dr. Chen Chaomei from Drexel University. It can explain the structure, patterns, and distribution of scientific knowledge, producing the so called “scientific knowledge graph”[1]. CiteSpace is mainly used to organize theoretical viewpoints, track evolutionary paths, predict development trends, conduct in-depth research on academic history, and identify current research hotspots within a particular field[1]. It is a practical quantitative analysis tool used to scrutinize research documents[2].

CiteSpace analysis methods include co-citation analysis, co-occurrence analysis, burst detection, and cluster analysis[1]. Co-citation analysis consists of examining the relationship between two studies based on their co-citation in the third study. Studies that are frequently cited together are considered to be more similar and interrelated. Co-occurrence analysis the number of times that a particular keyword appears in the literature of a given field, and measures the correlation between them by co-occurrence. Burst detection can identify fluctuations in the use of particular keywords, while cluster analysis groups objects on the basis of their similarities, helping to analyze multiple clusters formed[3-6].

Centrality is a critical metric that gauges the importance of an object in a network. Nodes with an intermediate centrality value greater than 0.1 are called central nodes or key nodes, which have an important influence in the research field and are often act as bridges connecting different kinds of research objects like articles, keywords, and countries[7].

CiteSpace is uniquely positioned to pinpoint pivotal aspects and future directions in the field of research, making it an invaluable tool for scholarly investigations[1]. In this study, the bibliometrics analysis software CiteSpace was deployed to analyze the existing literature on AI in orthopedics. Critical readings were also conducted to delve into key research findings and provide vital insights into this subject matter.

**References**

1. Chen CM. CiteSpace II: Detecting and visualizing emerging trends and transient patterns in scientific literature. Journal of the American Society for Information Science and Technology 2006;**57**(3):359-77 doi: 10.1002/asi.20317.

2. Hood WW, Wilson CS. The literature of bibliometrics, scientometrics, and informetrics. Scientometrics 2001;**52**(2):291-314 doi: 10.1023/a:1017919924342.

3. Donthu N, Kumar S, Mukherjee D, Pandey N, Lim WM. How to conduct a bibliometric analysis: An overview and guidelines. Journal of Business Research 2021;**133**:285-96 doi: 10.1016/j.jbusres.2021.04.070.

4. Ding Y, Chowdhury GG, Foo S. Bibliometric cartography of information retrieval research by using co-word analysis. Information Processing & Management 2001;**37**(6):817-42 doi: 10.1016/s0306-4573(00)00051-0.

5. Su HN, Lee PC. Mapping knowledge structure by keyword co-occurrence: a first look at journal papers in Technology Foresight. Scientometrics 2010;**85**(1):65-79 doi: 10.1007/s11192-010-0259-8.

6. Liu ZG, Yin YM, Liu WD, Dunford M. Visualizing the intellectual structure and evolution of innovation systems research: a bibliometric analysis. Scientometrics 2015;**103**(1):135-58 doi: 10.1007/s11192-014-1517-y.

7. Wang LY, Zhao L, Mao GZ, Zuo J, Du HB. Way to accomplish low carbon development transformation: A bibliometric analysis during 1995-2014. Renewable & Sustainable Energy Reviews 2017;**68**:57-69 doi: 10.1016/j.rser.2016.08.021.
